# Supplementary material for: RAF inhibitors promote RAS-RAF interaction by allosterically disrupting RAF autoinhibition
Source: Nat Commun. 2017 Oct 31;8:1211. doi: 10.1038/s41467-017-01274-0 (PMC5662619; doi:10.1038/s41467-017-01274-0)

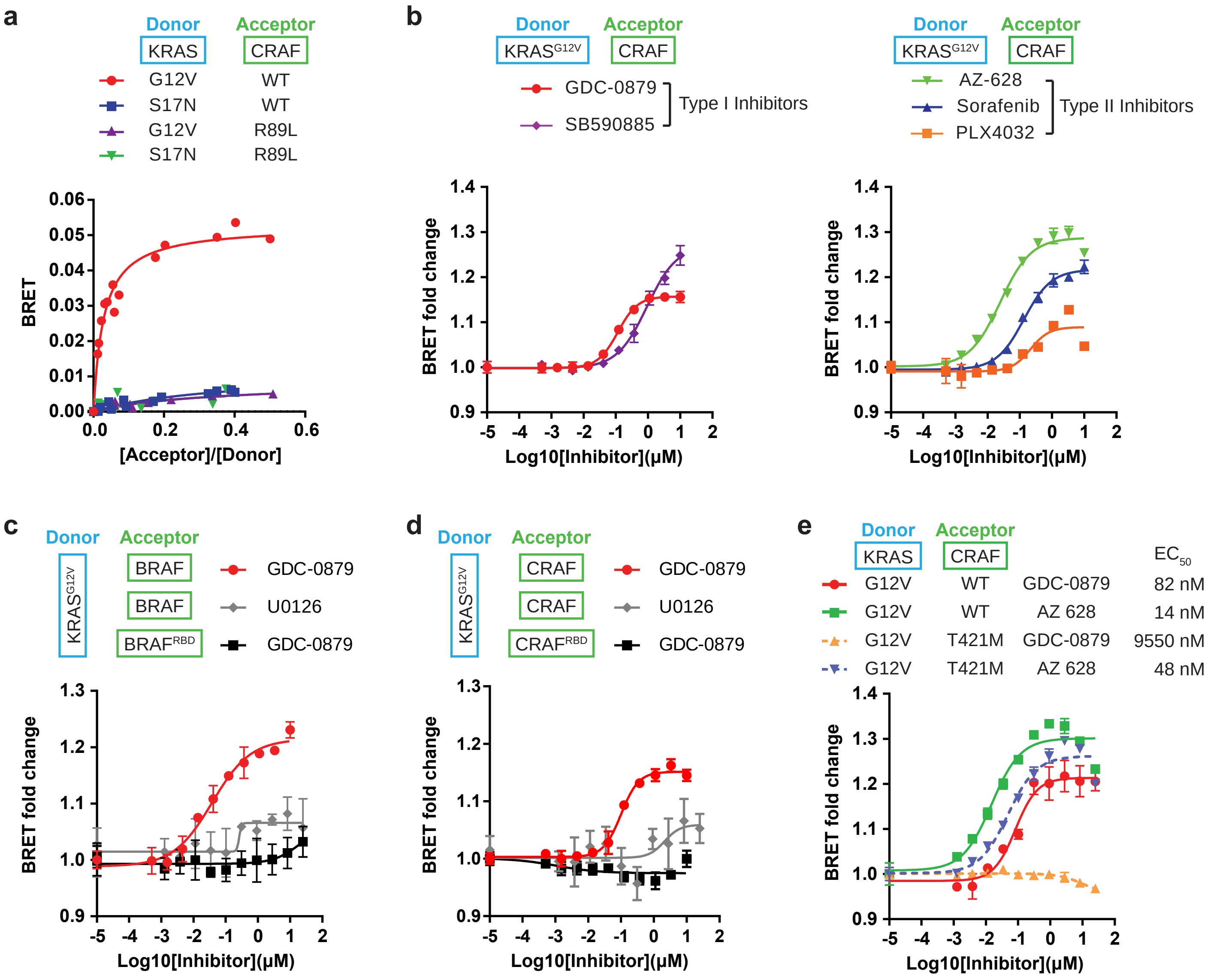

Supplementary Figure 1 | RAF inhibitors promote RAS-RAF complex formation as determined by BRET assays. (**a**) BRET titration curves of KRAS-CRAF biosensors. The RlucII (donor) and GFP10 (acceptor) moieties were inserted at the N-terminus of KRAS and CRAF variants, respectively. (**b**) Dose response curves performed on cells transfected with $KRAS^{G12V}$ and CRAF BRET probes. The left panel shows BRET fold changes with Type I inhibitor treatment, while the right panel shows treatment with Type II inhibitors. (**c**) and (**d**) MEK inhibition with U0126 has marginal impact on the BRET signals produced by the $KRAS^{G12V}$-BRAF and $KRAS^{G12V}$-CRAF BRET probes. Additionally, GDC-0879 does not induce the association of $KRAS^{G12V}$ with the BRAF or CRAF RBD alone probes (black curves). As positive controls, GDC-0879 increased BRET signals from $KRAS^{G12V}$-BRAF or $KRAS^{G12V}$-CRAF (red curves). (**e**) The CRAF gatekeeper mutation (T421M) prevents the induction of the $KRAS^{G12V}$-CRAF interaction by GDC-0879, but not by AZ-628. EC_{50}s for each dose-response curve is indicated. Error bars in dose-response curves correspond to mean values ± s.d. of technical duplicates of a representative biological triplicate.

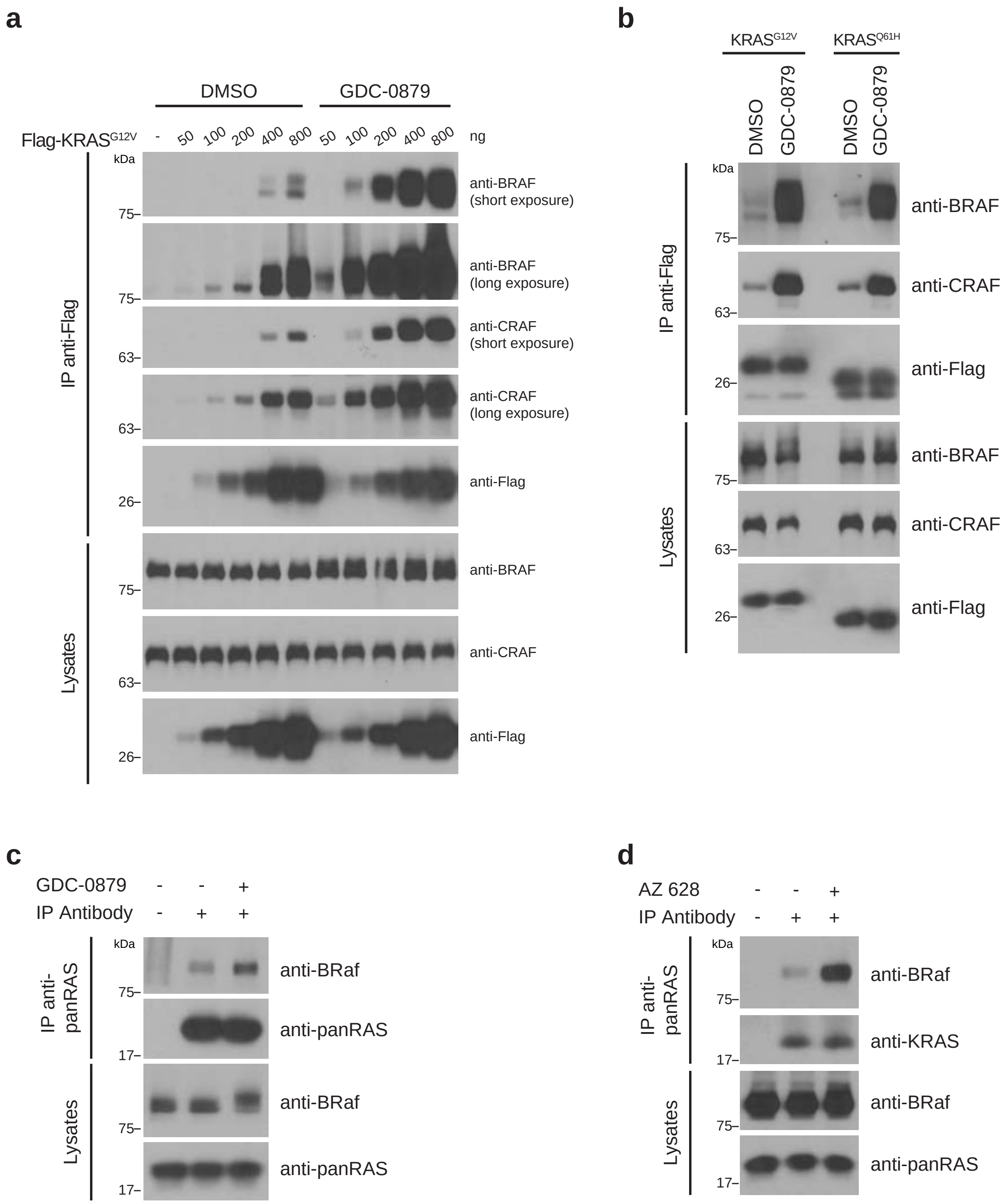

Supplementary Figure 2 | RAF inhibitors promote RAS-RAF complex formation as determined by co-IP. (**a**) HEK293T cells were transfected with the indicated increasing amounts of the Flag-KRAS^{G12V} construct. GDC-0879 treatment stimulated the interaction between Flag-KRAS^{G12V} and endogenous BRAF and CRAF in each condition. (**b**) As with KRAS^{G12V}, GDC-0879 induces the association of endogenous BRAF and CRAF with the KRAS^{Q61H} oncogenic allele. GDC-0879 (**c**) and AZ-628 (**d**) enhance the interaction between endogenous RAS and BRAF in Hela cells. Cell lysates were immunoprecipitated with an anti-panRAS antibody and probed for the indicated proteins. As negative controls, the first lane in (c) and (d) shows IP samples where the anti-panRAS antibody was omitted. Both compounds were tested at 10 µM.

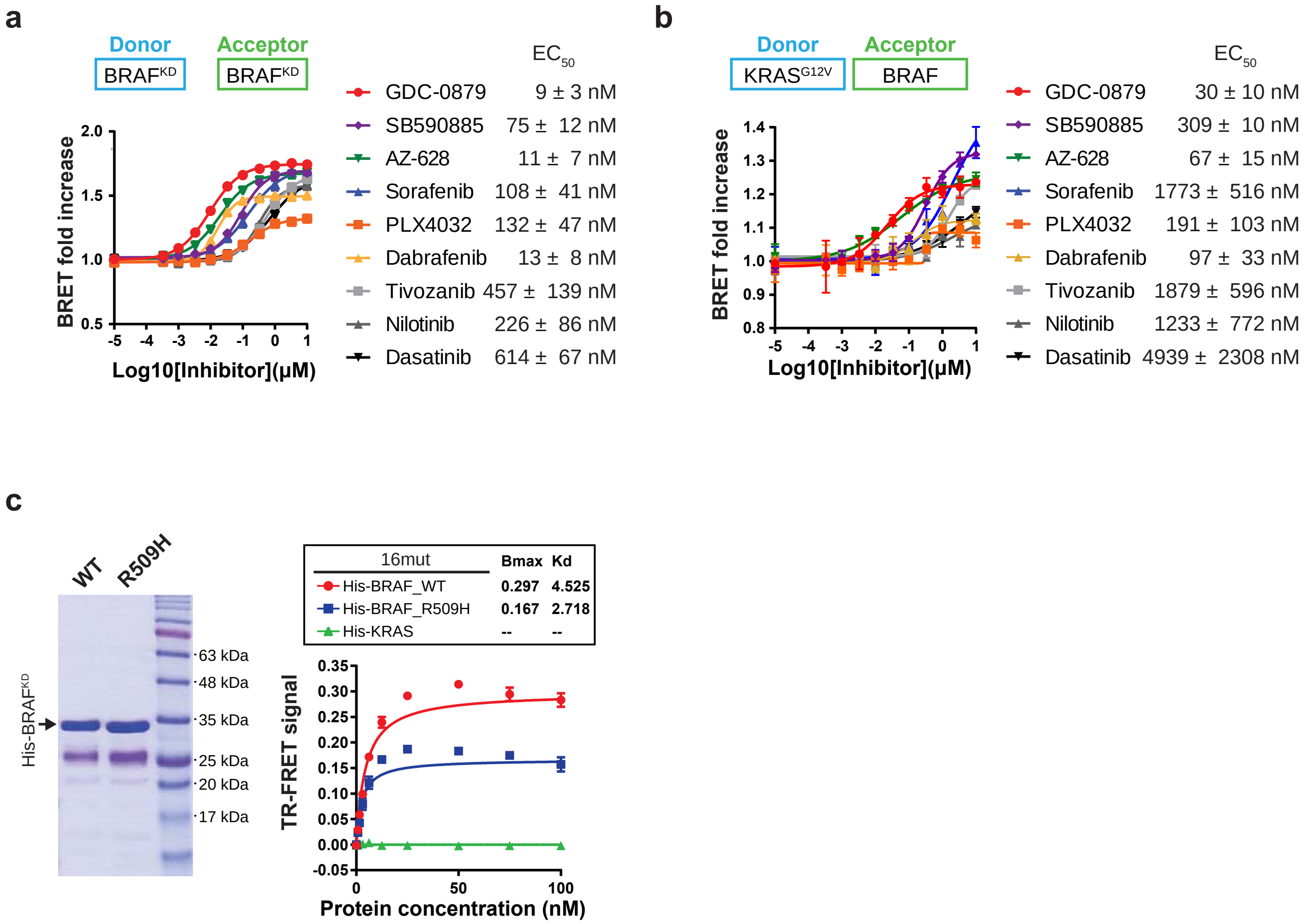

Supplementary Figure 3 | Compound-induced RAS-RAF association is connected to RAF dimerization. As measured by BRET, various RAF and off-target inhibitors stimulate BRAF kinase domain dimerization (**a**) as well as enhance $KRAS^{G12V}$-BRAF association (**b**). The EC_{50}s indicated are the average of at least three independent replicates (Supplementary Table 1). (**c**) Coomassie brilliant blue-stained gel showing purified His-tagged 16mut-$BRAF^{WT}$ and 16-mut$BRAF^{R509H}$. Saturation of the TR-FRET signal between LANCE® Europium-coupled anti-His antibody (Perkin Elmer) and the Alexa Fluor® 647-labeled kinase tracer 178 (Invitrogen). The curves were produced by titrating in WT or R509H His-tagged BRAF kinase domains. Saturation was not observed with the His-tagged KRAS negative control, which does not interact with the kinase tracer. Error bars in dose-response curves and the saturation curve correspond to mean values ± s.d. of technical duplicates of a representative biological triplicate.

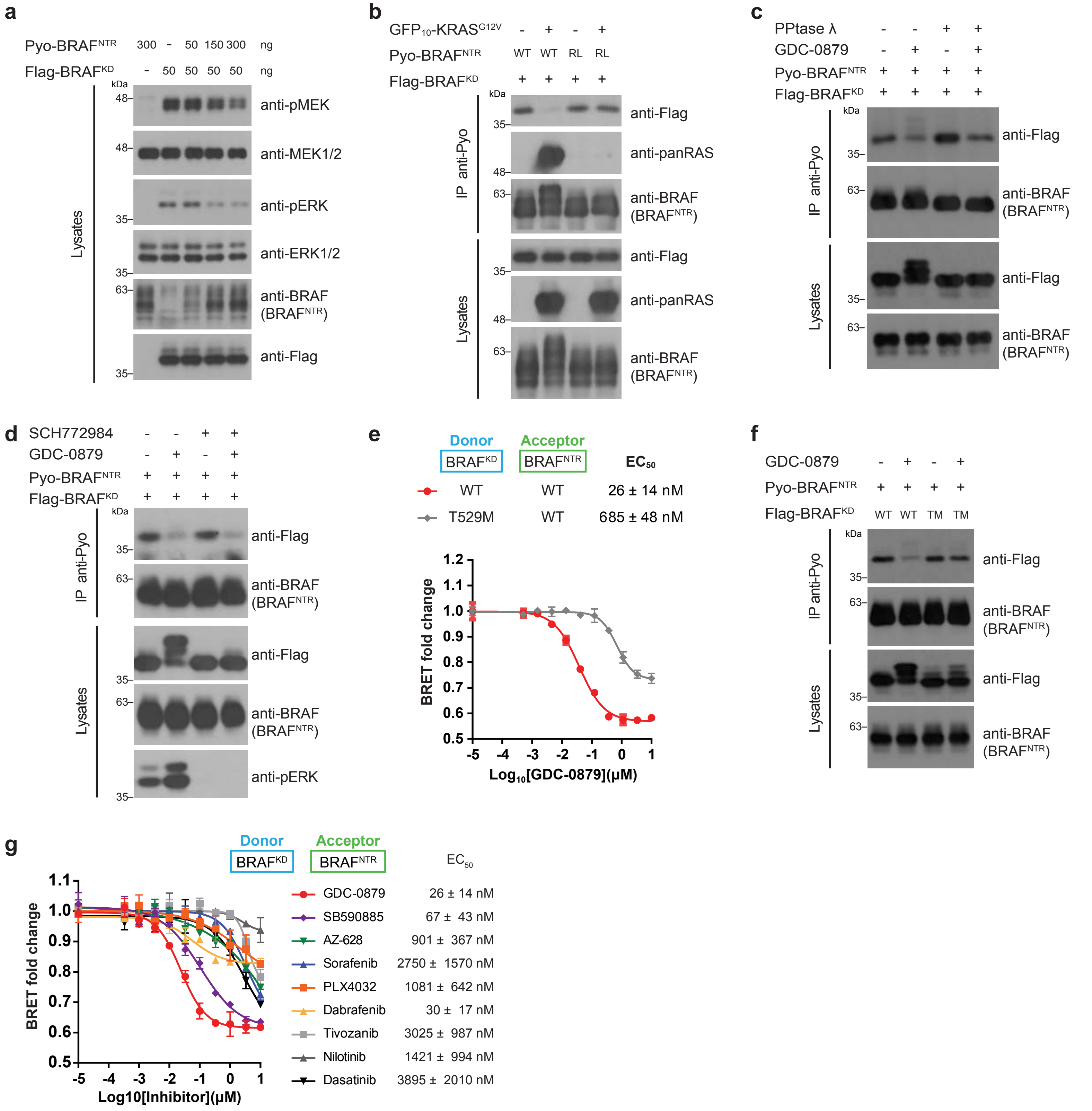

Supplementary Figure 4 | RAF inhibitors disrupt BRAF autoinhibition. (**a**) The catalytic activity of the BRAF kinase domain is inhibited by co-expressing the $BRAF^{NTR}$ in a dose-dependent manner. The amounts of transfected plasmids (ng) are indicated and cell lysates were probed with the indicated antibodies. Anti-BRAF was used to detect Pyo-tagged $BRAF^{NTR}$. (**b**) GFP10-$KRAS^{G12V}$ binds to $BRAF^{NTR}$ and thereby abrogates the NTR-KD interaction. A $BRAF^{NTR}$ comprising the R188L (RL) RBD mutation is insensitive to $KRAS^{G12V}$-induced disruption of the NTR-KD interaction. Cell lysates were immunoprecipitated with anti-Pyo and probed with the indicated antibodies. (**c**) PPtase λ treatment eliminates the mobility shift of the BRAF kinase domain induced by GDC-0879, but did not alter the ability of GDC-0879 to disrupt the NTR-KD complex formation. 1 μM of GDC-0879 was used. (**d**) The SCH772984 ERK inhibitor prevents the mobility shift of the BRAF kinase domain induced by GDC-0879, but did not alter the ability of GDC-0879 to disrupt the NTR-KD interaction. GDC-0879 and SCH772984 were used at 1 and 2 μM, respectively. Cell lysates were immunoprecipitated with anti-Pyo and probed with the indicated antibodies. The BRAF gatekeeper mutation T529M (TM) reduced the potency of GDC-0879 to disrupt the BRAF intramolecular interaction as measured by BRET (**e**) and co-IP (**f**). (**g**) $BRAF^{NTR}$-$BRAF^{KD}$ BRET dose-response curves for a panel of RAF inhibitors. EC_{50}s are the average of at least three independent repeats. Error bars in dose-response curves correspond to mean values ± s.d. of technical duplicates of a representative biological triplicate.

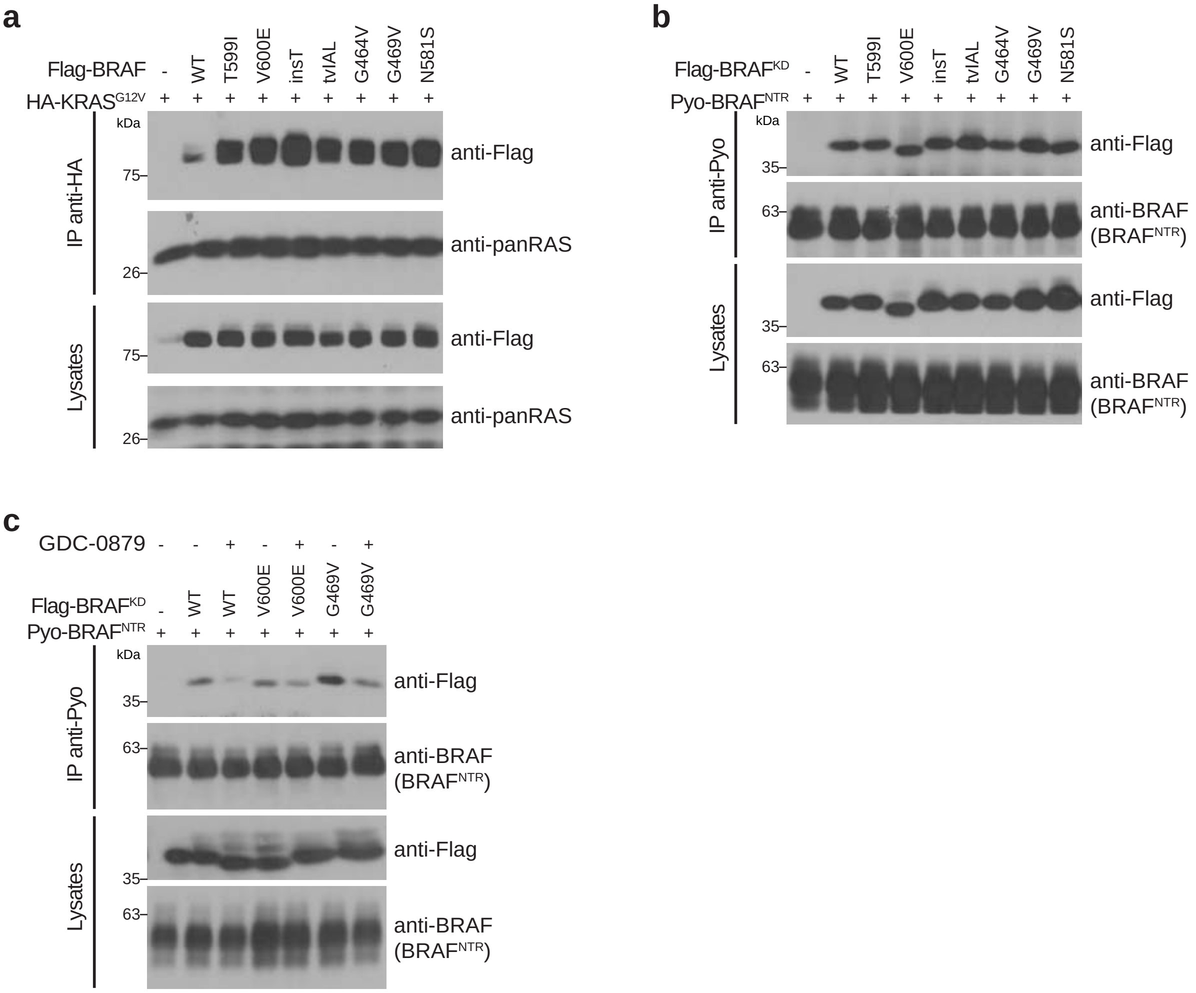

Supplementary Figure 5 | Effect of BRAF oncogenic alleles on RAS-RAF association and NTR-KD interaction. (a) BRAF oncogenic alleles associate to a greater extent to KRAS^{G12V} compared to WT BRAF as determined by co-IP. (**b**) BRAF oncogenic mutations do not alter the NTR-KD association. (**c**) GDC-0879 treatment (10 μM) disrupts the NTR-KD interactions observed for two oncogenic variants, namely, the V600E or G469V mutants. Treatment with the ERK inhibitor SCH772984 (2 μM) was used in (b) and (c) to eliminate band shifting of BRAF kinase domain induced by ERK-mediated negative feedback.

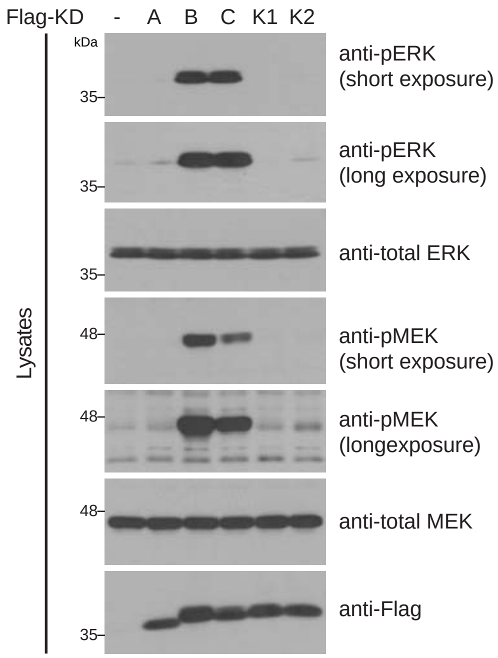

Supplementary Figure 6 | Kinase activity associated to the five RAF family members. Phosphorylation of MEK and ERK was used as a proxy to evaluate the relative catalytic activity of ARAF, BRAF, and CRAF as well as KSR1 and KSR2 kinase domains in transfected HEK293T cells. BRAF and CRAF kinase domains were highly active, whereas the three other kinase domains did not stimulate MEK or ERK phosphorylation.

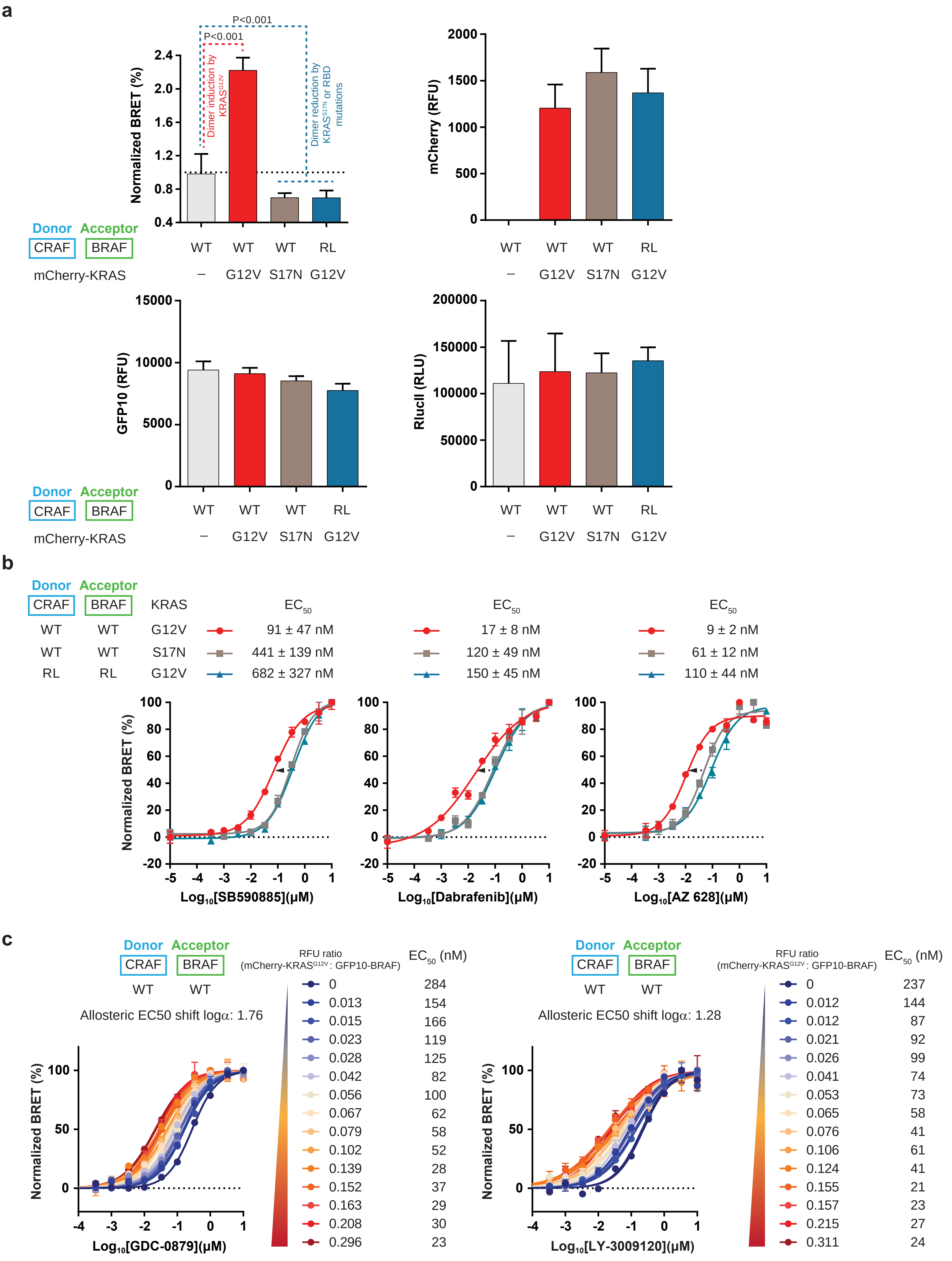

a
P<0.001
P<0.001
Dimer induction by KRAS^{G12V}
Dimer reduction by KRAS^{S17N} or RBD mutations
Normalized BRET (%)
mCherry (RFU)
GFP10 (RFU)
Rlucll (RLU)
Donor Acceptor
CRAF BRAF
mCherry-KRAS
WT WT WT RL
– G12V S17N G12V
b
Donor Acceptor
CRAF BRAF KRAS
WT WT G12V
WT WT S17N
RL RL G12V
EC$_{50}$
91 ± 47 nM
441 ± 139 nM
682 ± 327 nM
EC$_{50}$
17 ± 8 nM
120 ± 49 nM
150 ± 45 nM
EC$_{50}$
9 ± 2 nM
61 ± 12 nM
110 ± 44 nM
Normalized BRET (%)
Log$_{10}$[SB590885](μM)
Log$_{10}$[Dabrafenib](μM)
Log$_{10}$[AZ 628](μM)
c
Donor Acceptor
CRAF BRAF
WT WT
Allosteric EC50 shift logα: 1.76
RFU ratio (mCherry-KRAS^{G12V} : GFP10-BRAF)
EC$_{50}$ (nM)
0 284
0.013 154
0.015 166
0.023 119
0.028 125
0.042 82
0.056 100
0.067 62
0.079 58
0.102 52
0.139 28
0.152 37
0.163 29
0.208 30
0.296 23
Normalized BRET (%)
Log$_{10}$[GDC-0879](μM)
Donor Acceptor
CRAF BRAF
WT WT
Allosteric EC50 shift logα: 1.28
RFU ratio (mCherry-KRAS^{G12V} : GFP10-BRAF)
EC$_{50}$ (nM)
0 237
0.012 144
0.012 87
0.021 92
0.026 99
0.041 74
0.053 73
0.065 58
0.076 41
0.106 61
0.124 41
0.155 21
0.157 23
0.215 27
0.311 24
Normalized BRET (%)
Log$_{10}$[LY-3009120](μM)

Supplementary Figure 7 | ON-state RAF inhibitors promote BRAF-CRAF dimerization independently of RAS activity. (**a**) $KRAS^{G12V}$ stimulates full length BRAF-CRAF dimerization as measured by BRET. In contrast, expression of $KRAS^{S17N}$ or RAF probes bearing RBD mutations ($CRAF^{R89L}$ or $BRAF^{R188L}$; RL) decrease BRAF-CRAF BRET signal. Expression levels of mCherry, GFP10 and RlucII constructs are indicated for each condition. Error bars correspond to mean values ± s.d. of three independent transfections. (**b**) Three ON-state RAF inhibitors (SB590885, dabrafenib and AZ-628) induce full length BRAF-CRAF dimerization in a RAS-independent manner. CRAF-BRAF dimerization was measured by BRET in the presence of dominant-negative $KRAS^{S17N}$ (gray) or RBD-mutated $BRAF^{R188L}$ and $CRAF^{R89L}$ (RL; blue). Expression of active $KRAS^{G12V}$ potentiated the effect of both compounds on BRAF-CRAF dimerization (red series; dashed arrow). The calculated EC_{50}s are the average of at least three independent repeats. (**c**) Increasing expression of $KRAS^{G12V}$ progressively improves GDC-0879 and LY-3009120 potencies at inducing full length CRAF-BRAF dimerization. Expression of $KRAS^{G12V}$ relative to the RAF BRET probe was estimated as a ratio of mCherry-$KRAS^{G12V}$ (RFU) / GFP10-BRAF (RFU). This latter value was used as a proxy for allosteric modulator concentration to calculate the logα cooperativity factor using Graphpad Prism 6.07 built in “Allosteric EC_{50} shift” analysis. To facilitate comparison between conditions, the range between minimal and maximal BRET signals was normalized to 100%. Error bars in dose-response curves correspond to mean values ± s.d. of technical duplicates of a representative biological triplicate.

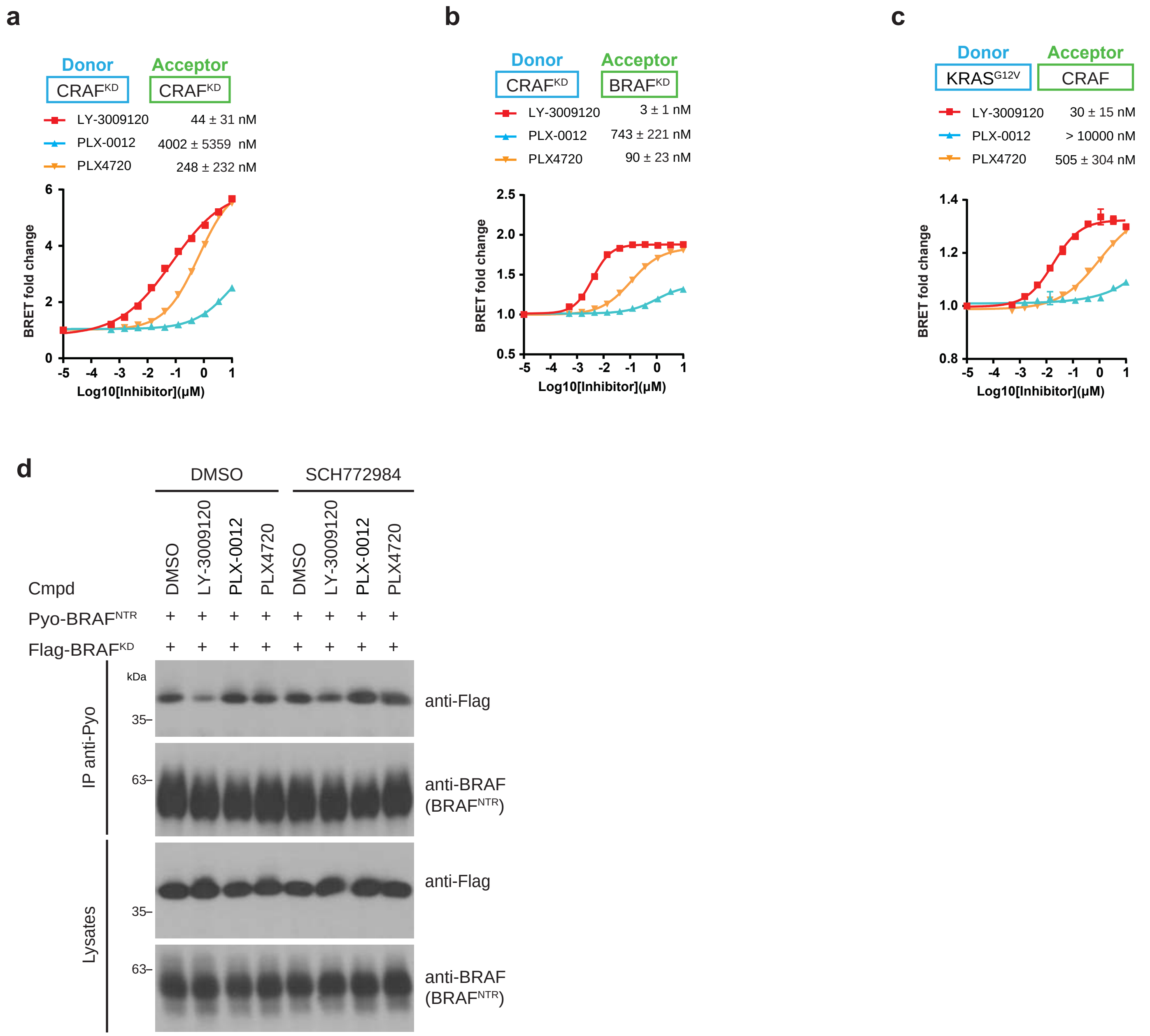

Supplementary Figure 8 | Mechanistic profiling of next-generation RAF inhibitors. BRET dose response curves show the differential potency of LY-3009120, PLX-0012, and PLX4720 at inducing CRAF-CRAF (**a**) and BRAF-CRAF (**b**) kinase domain dimers, and KRAS^{G12V}-CRAF association (**c**). The calculated EC_{50}s are the average of at least three independent repeats. Error bars in dose-response curves correspond to mean values ± s.d. of technical duplicates of a representative biological triplicate. (**d**) Effects of LY-3009120, PLX-0012, and PLX4720 on BRAF intramolecular interaction as determined by co-IP. 2 µM of SCH772984 was used to assess the impact of ERK negative feedback on the interaction. Cells were treated with 10 µM of each indicated RAF inhibitor.

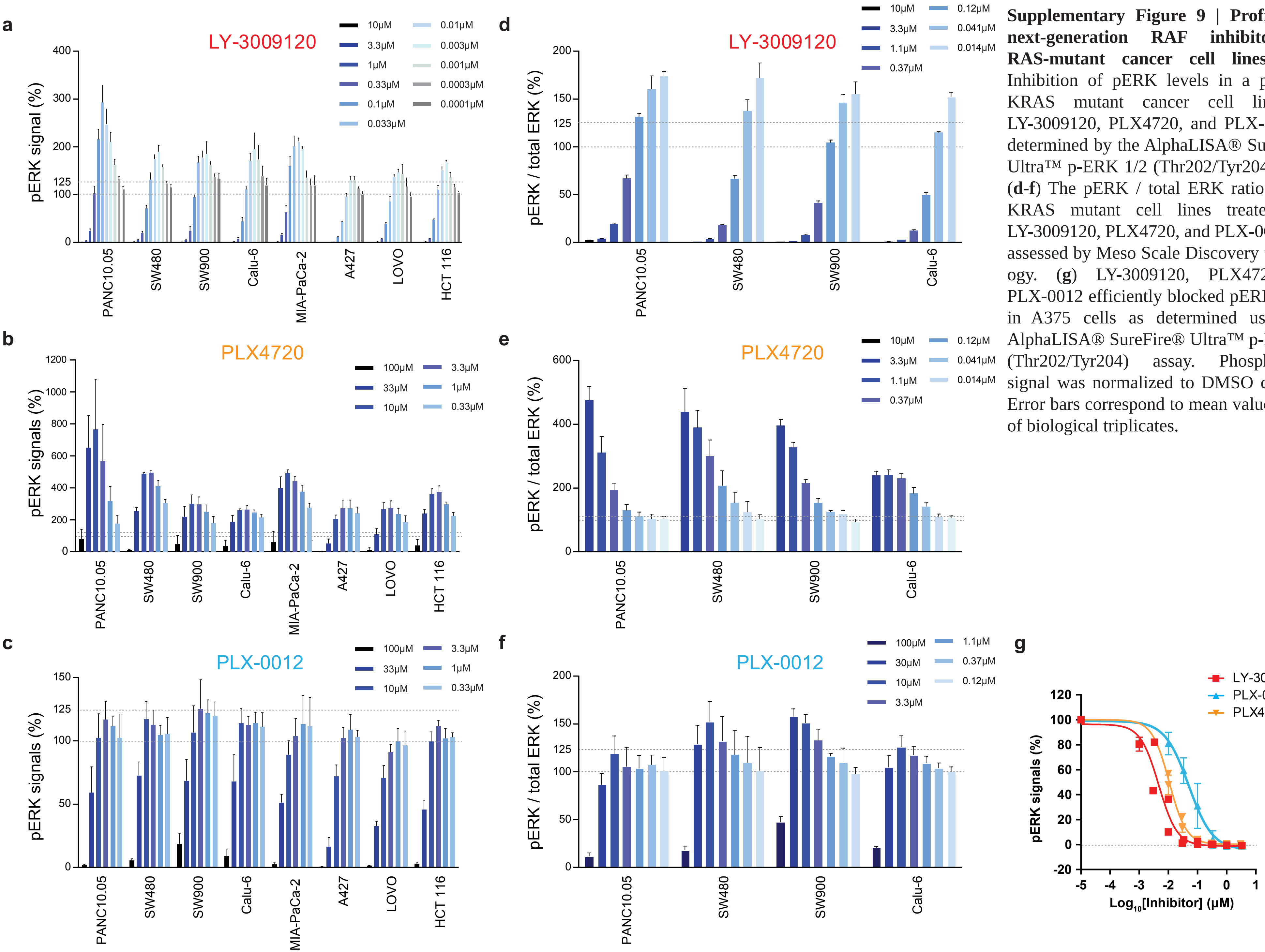

Supplementary Figure 9 | Profiling of next-generation RAF inhibitors in RAS-mutant cancer cell lines. **(a-c)** Inhibition of pERK levels in a panel of KRAS mutant cancer cell lines by LY-3009120, PLX4720, and PLX-0012 as determined by the AlphaLISA® SureFire® Ultra™ p-ERK 1/2 (Thr202/Tyr204) assay. **(d-f)** The pERK / total ERK ratio in four KRAS mutant cell lines treated with LY-3009120, PLX4720, and PLX-0012 was assessed by Meso Scale Discovery technology. **(g)** LY-3009120, PLX4720 and PLX-0012 efficiently blocked pERK signal in A375 cells as determined using the AlphaLISA® SureFire® Ultra™ p-ERK1/2 (Thr202/Tyr204) assay. Phospho-ERK signal was normalized to DMSO controls. Error bars correspond to mean values ± s.d. of biological triplicates.

Supplementary Figure 10. Uncropped western blots from primary figures are shown thereafter.

Uncropped immunoblots related to Fig. 1d

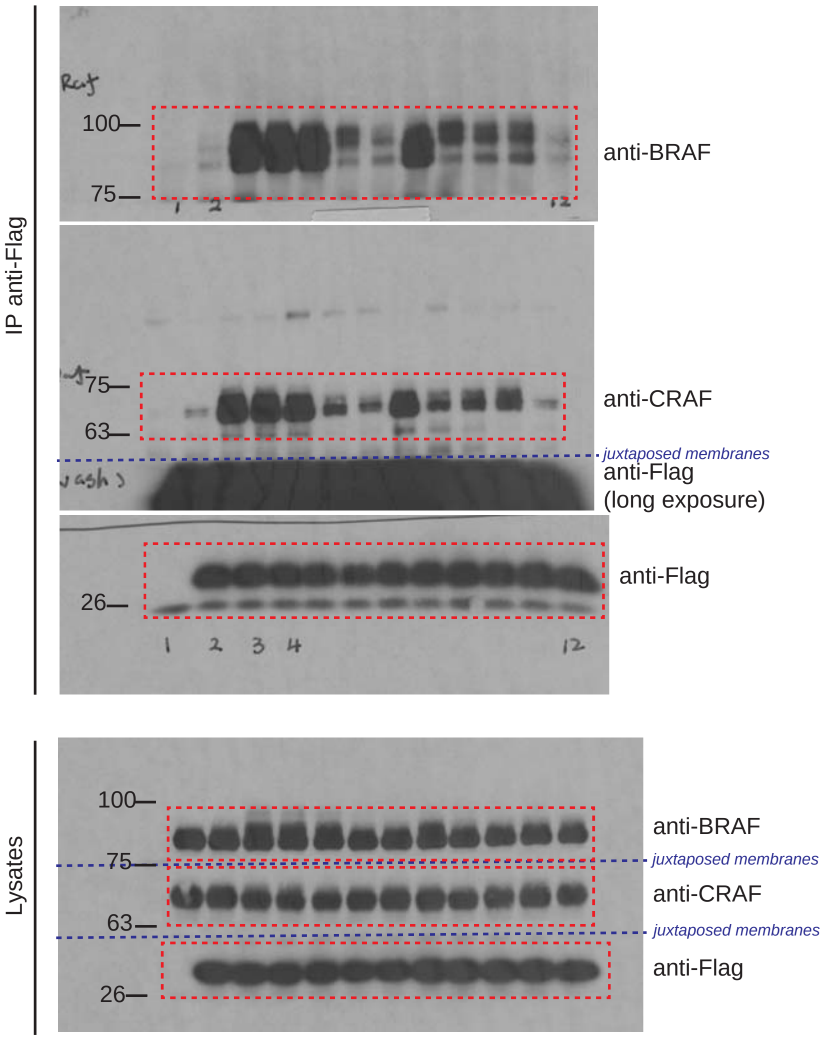

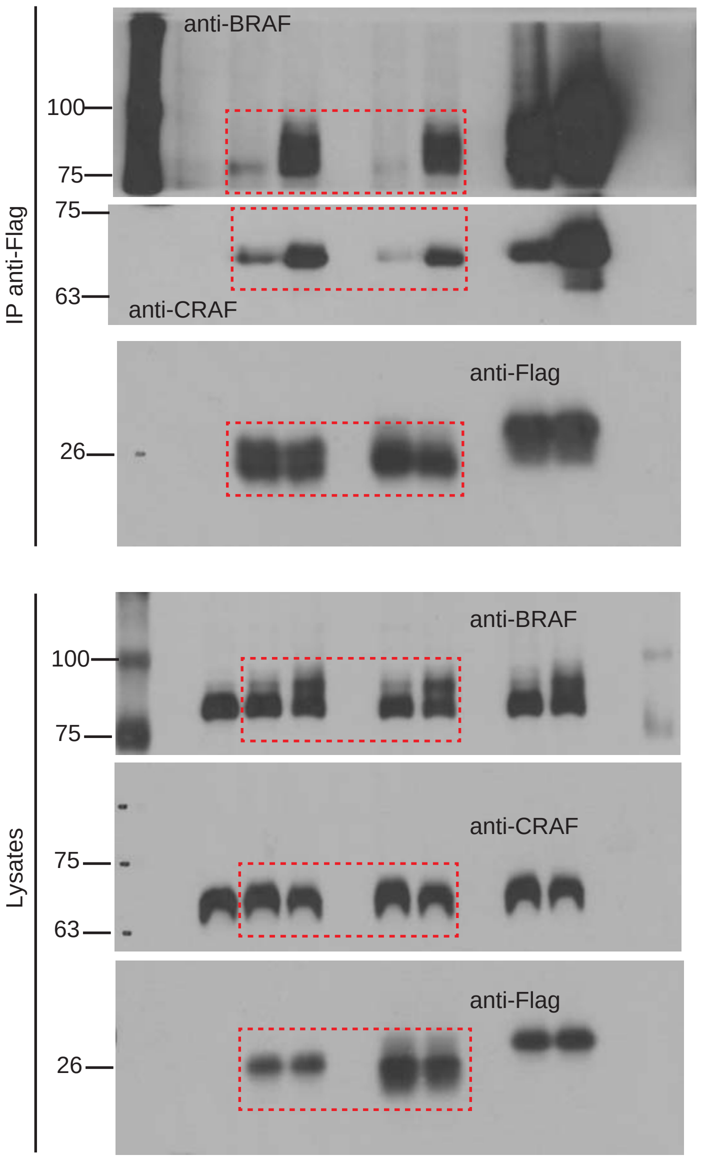

Uncropped immunoblots related to Fig. 1e

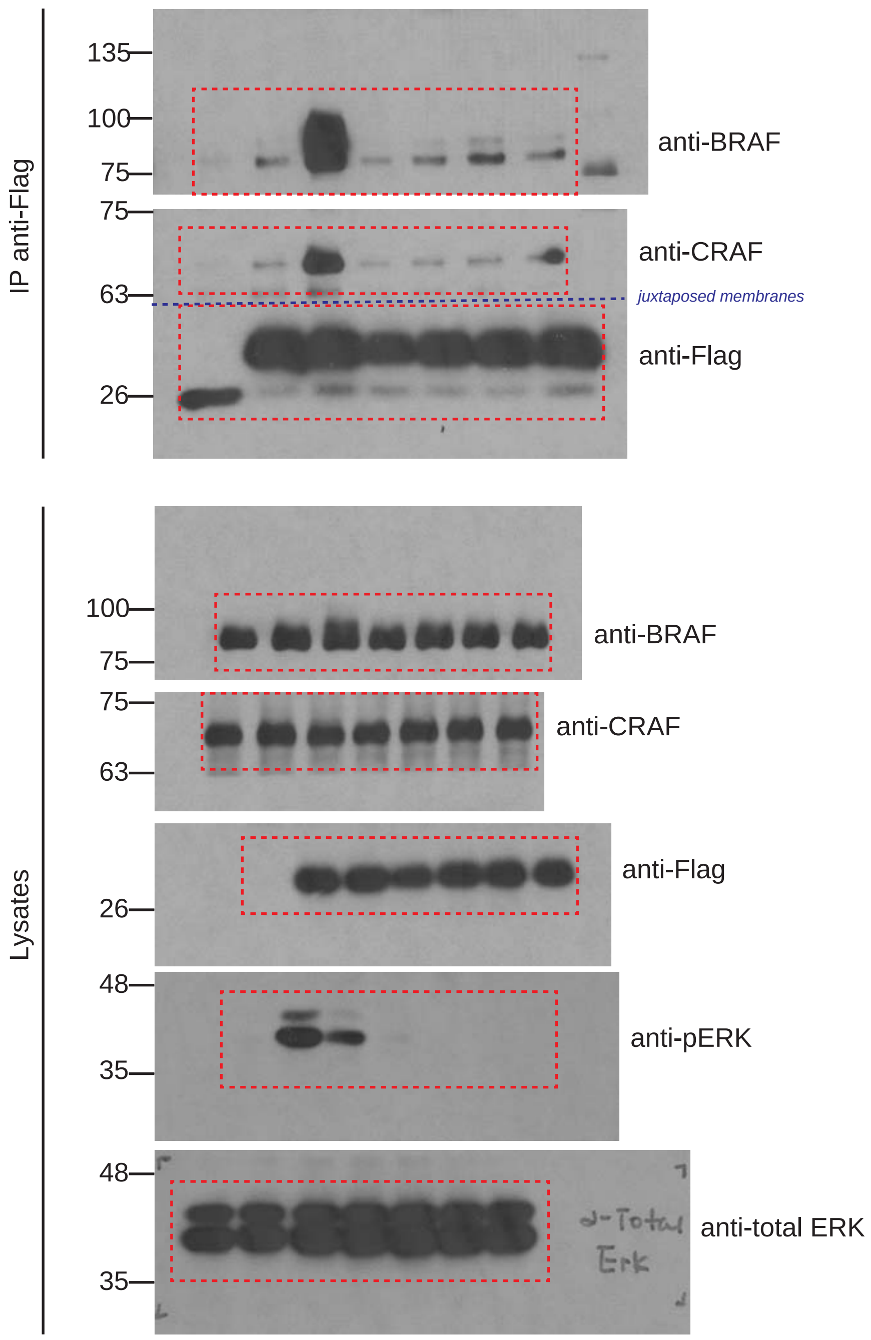

Uncropped immunoblots related to Fig. 2a

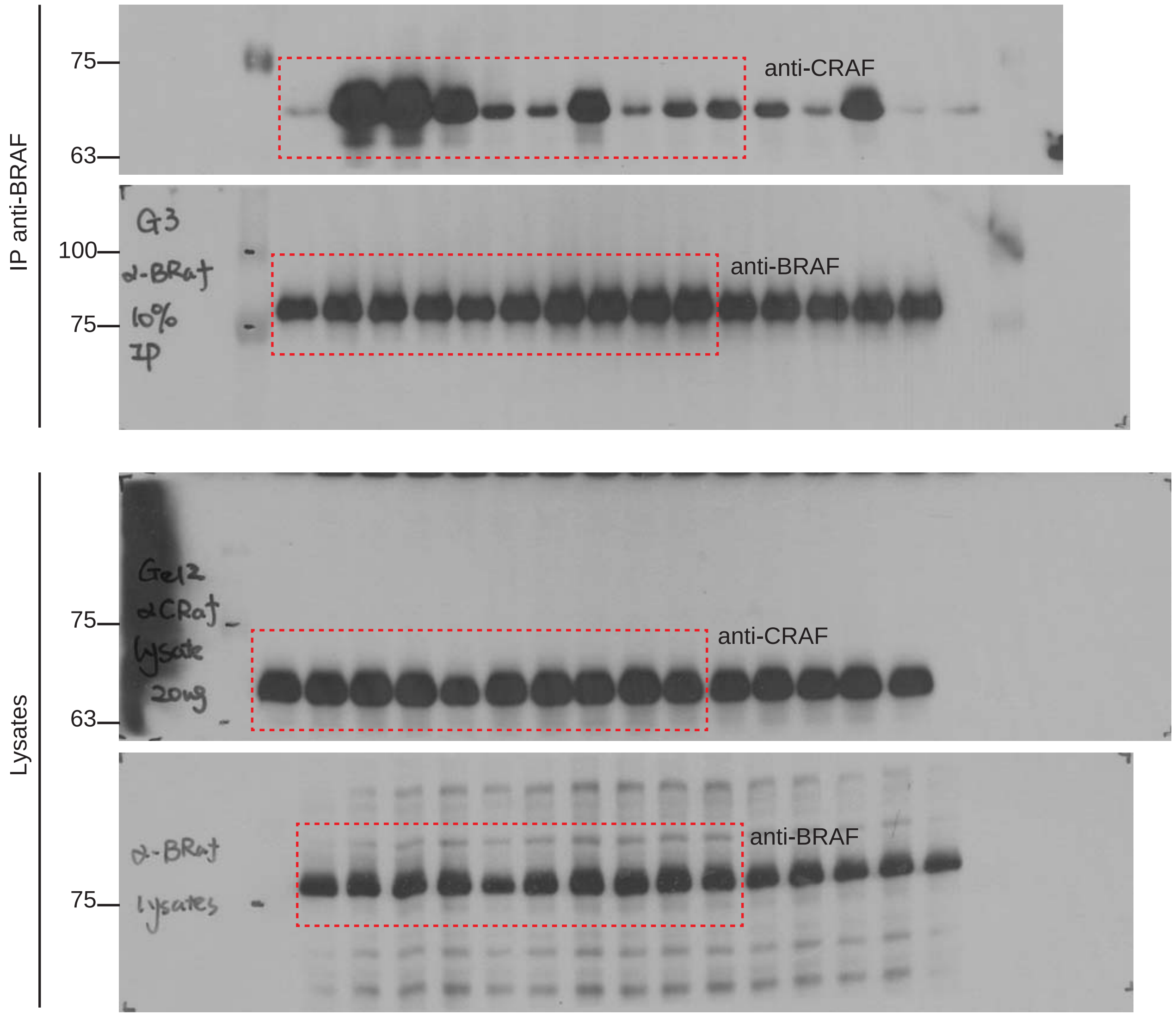

Uncropped immunoblots related to Fig. 2f

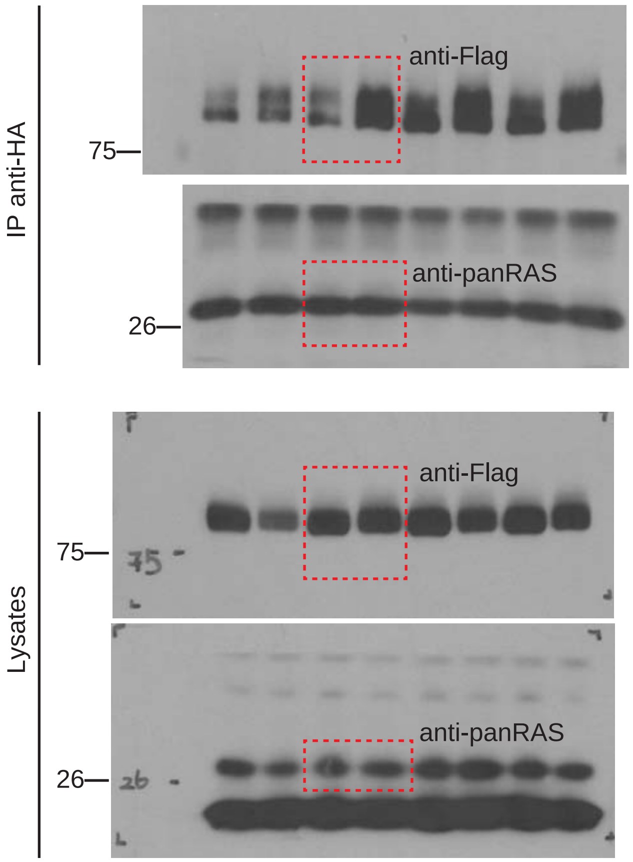

Uncropped immunoblots related to Fig. 3c

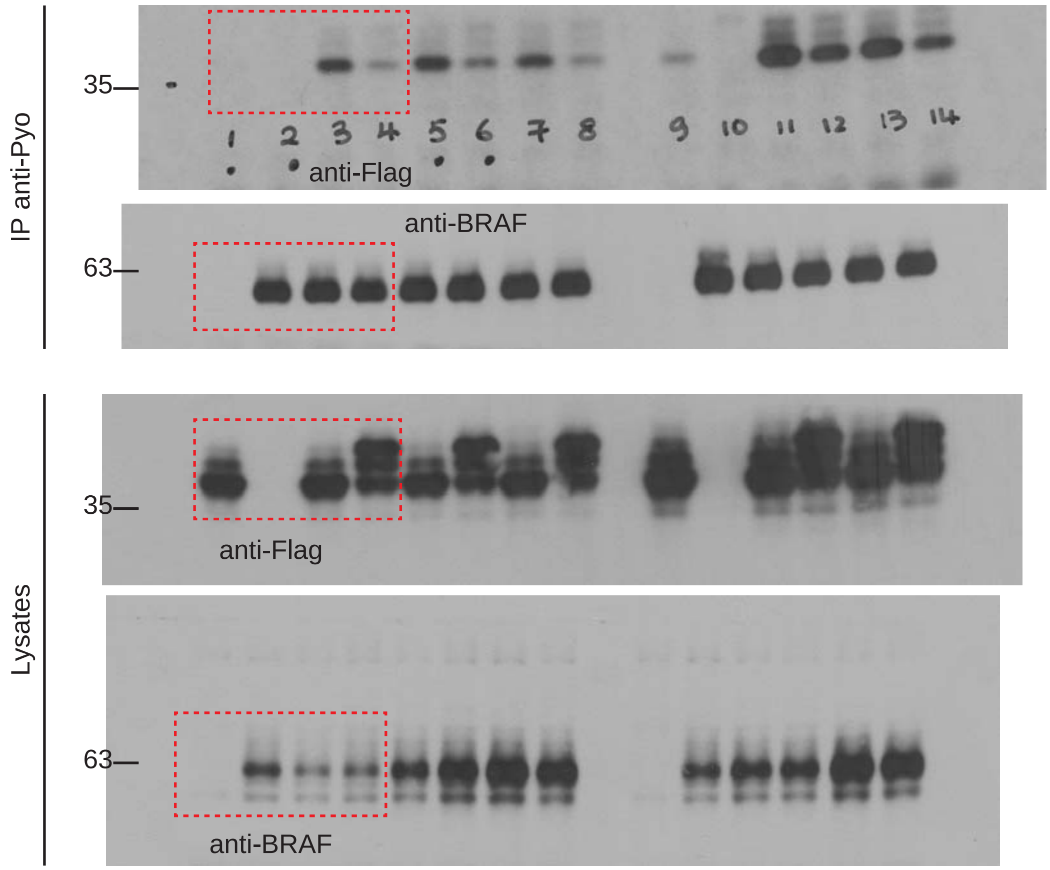

Uncropped immunoblots related to Fig. 3e

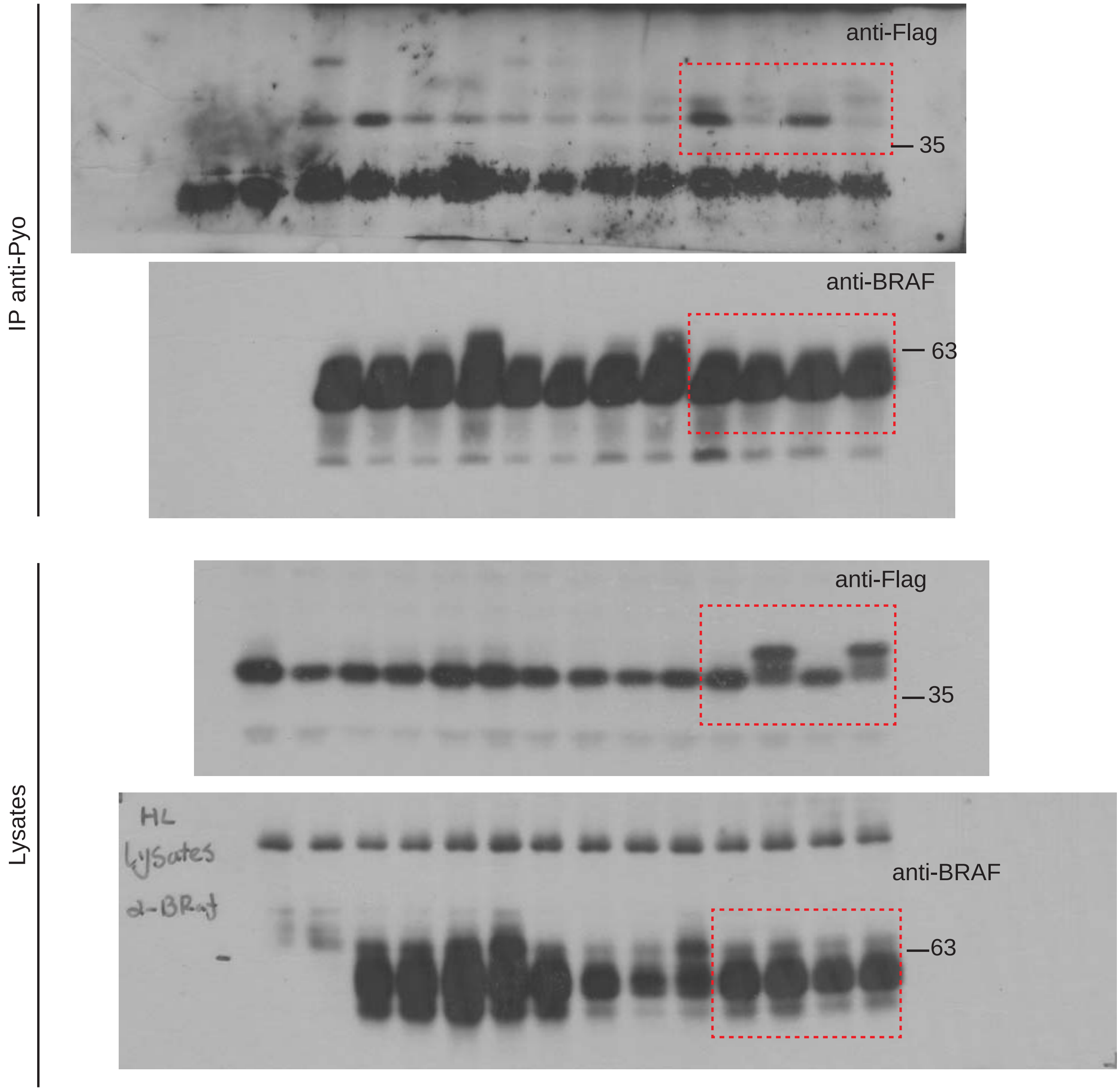

Uncropped immunoblots related to Fig. 4a

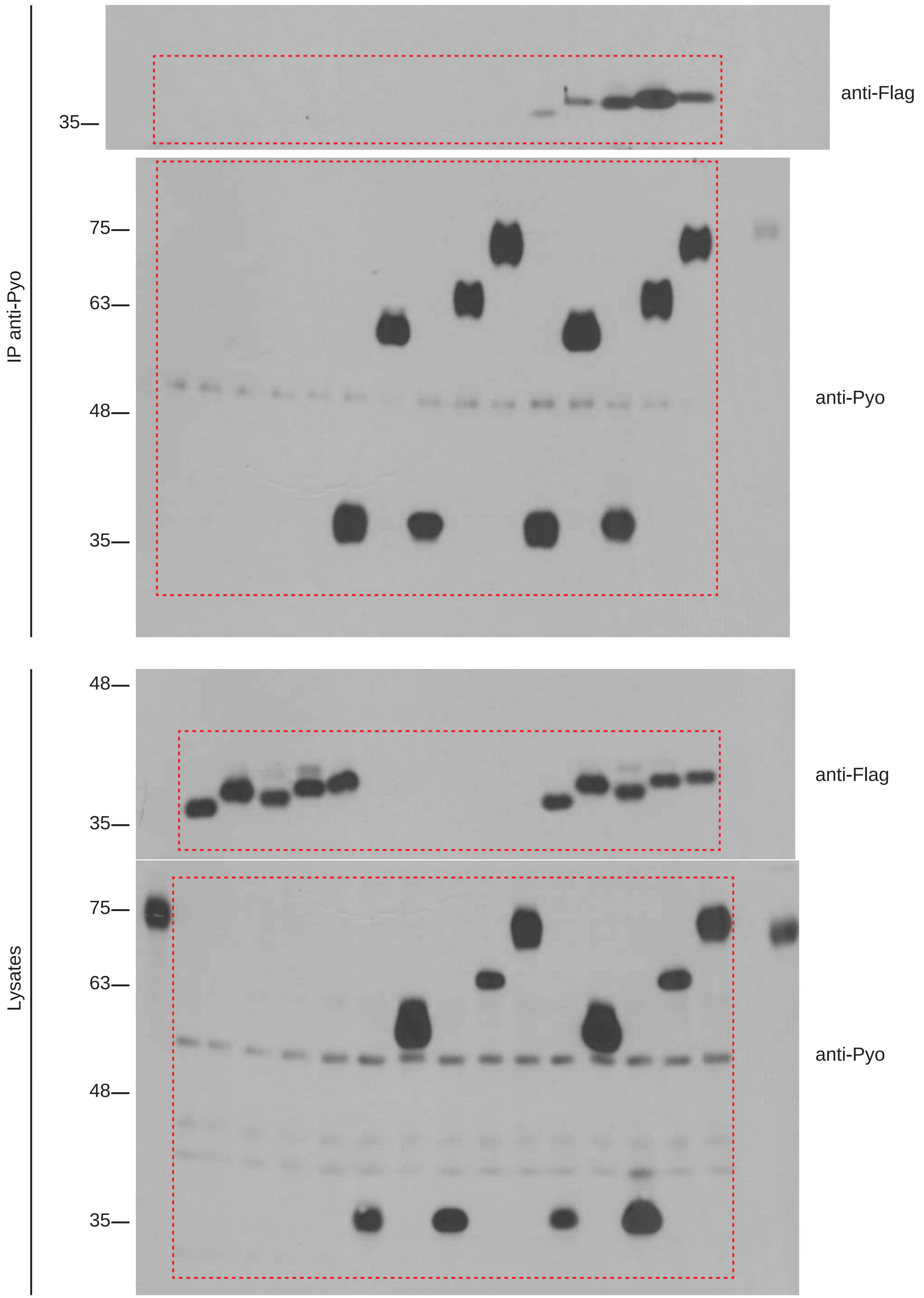

Uncropped immunoblots related to Fig. 4b

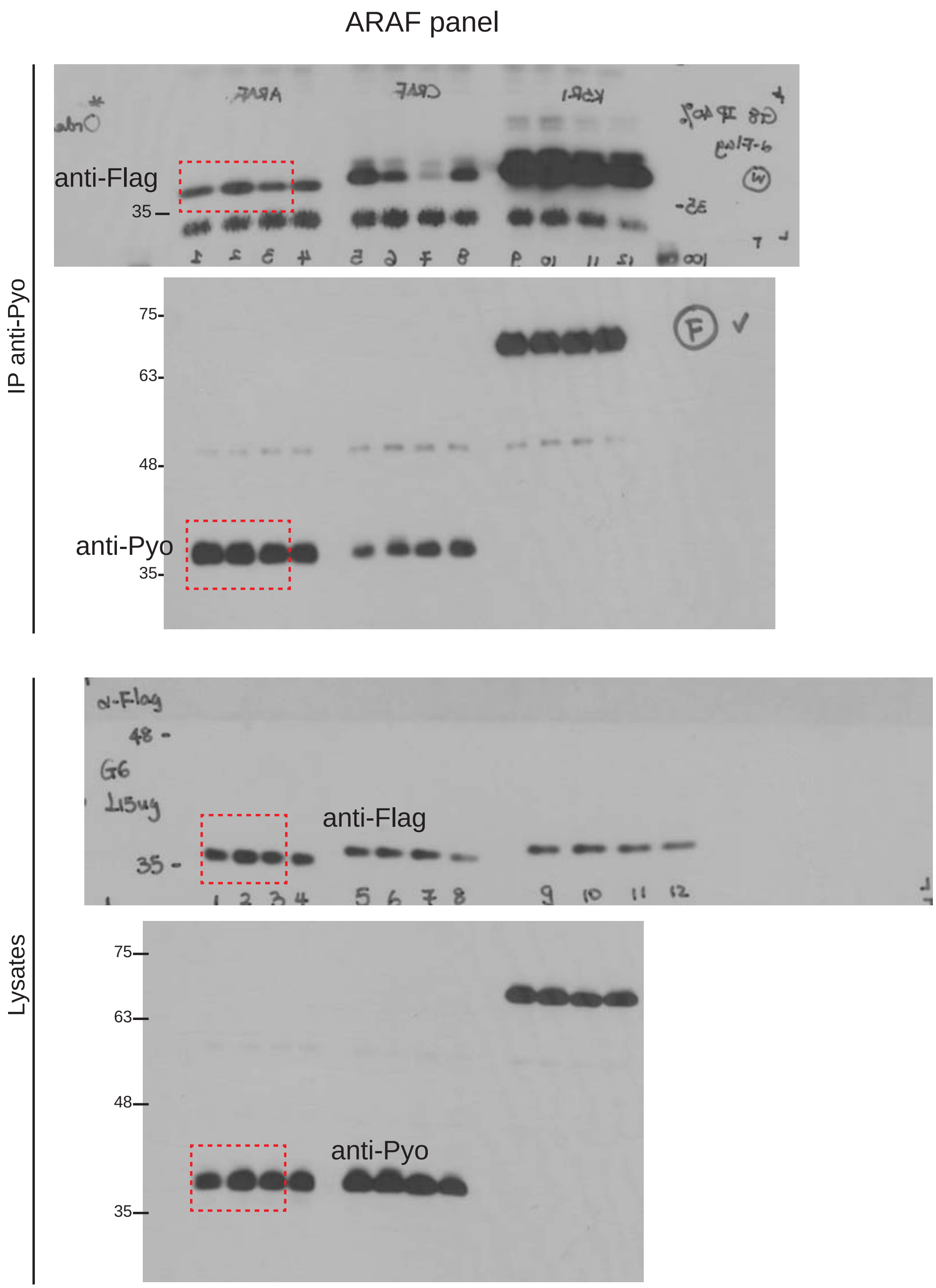

Uncropped immunoblots related to Fig. 4b

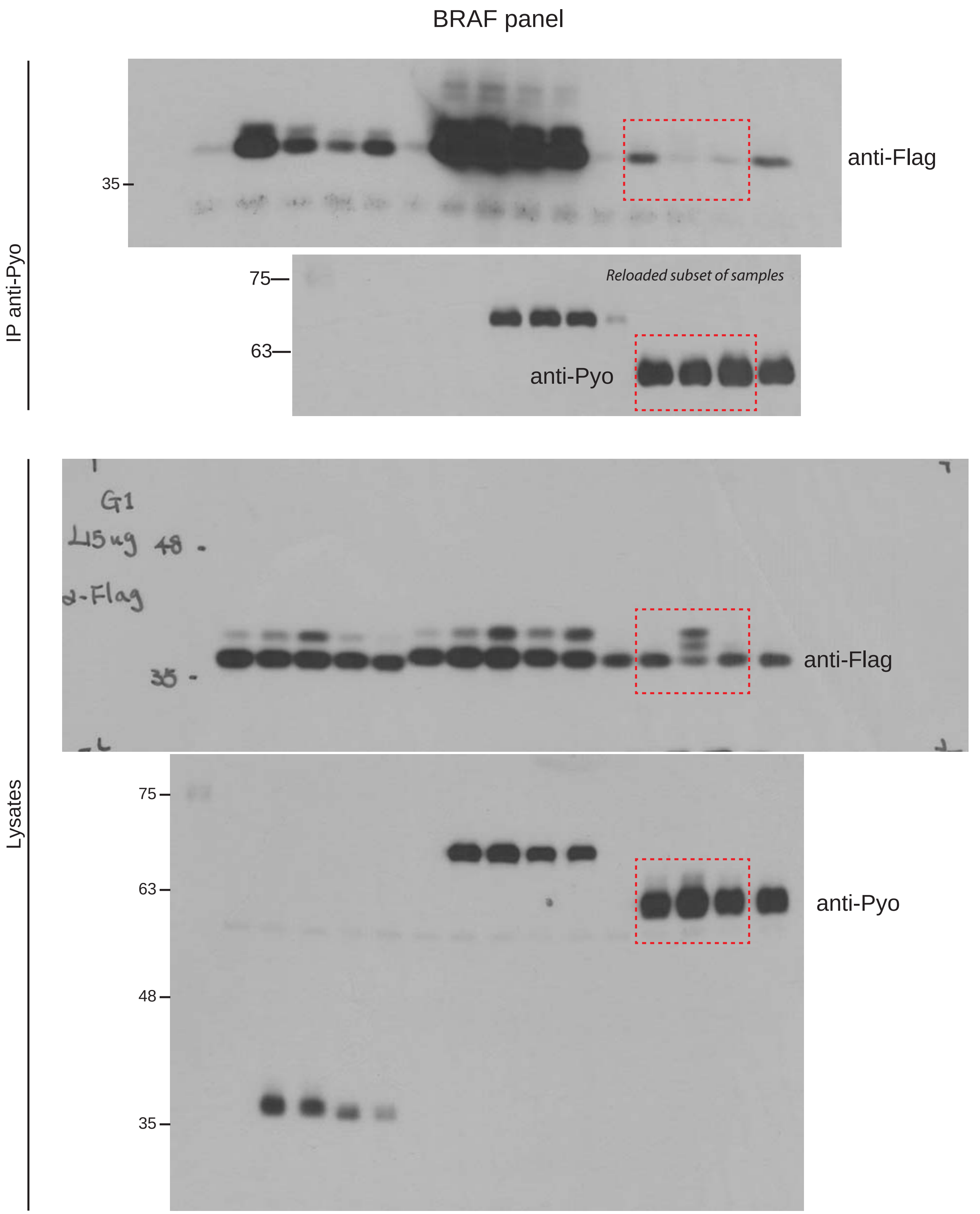

Uncropped immunoblots related to Fig. 4b

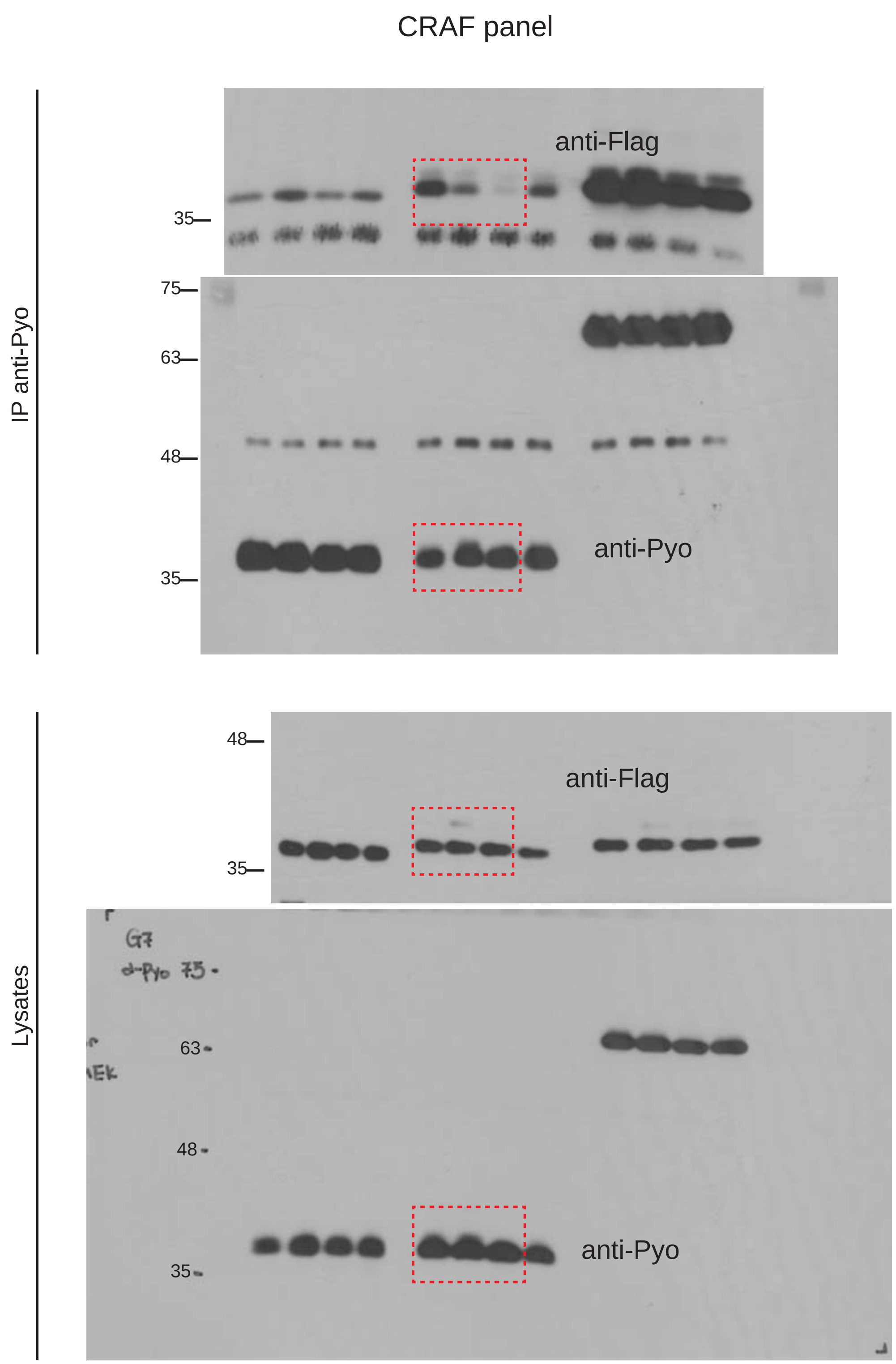

Uncropped immunoblots related to Fig. 4b

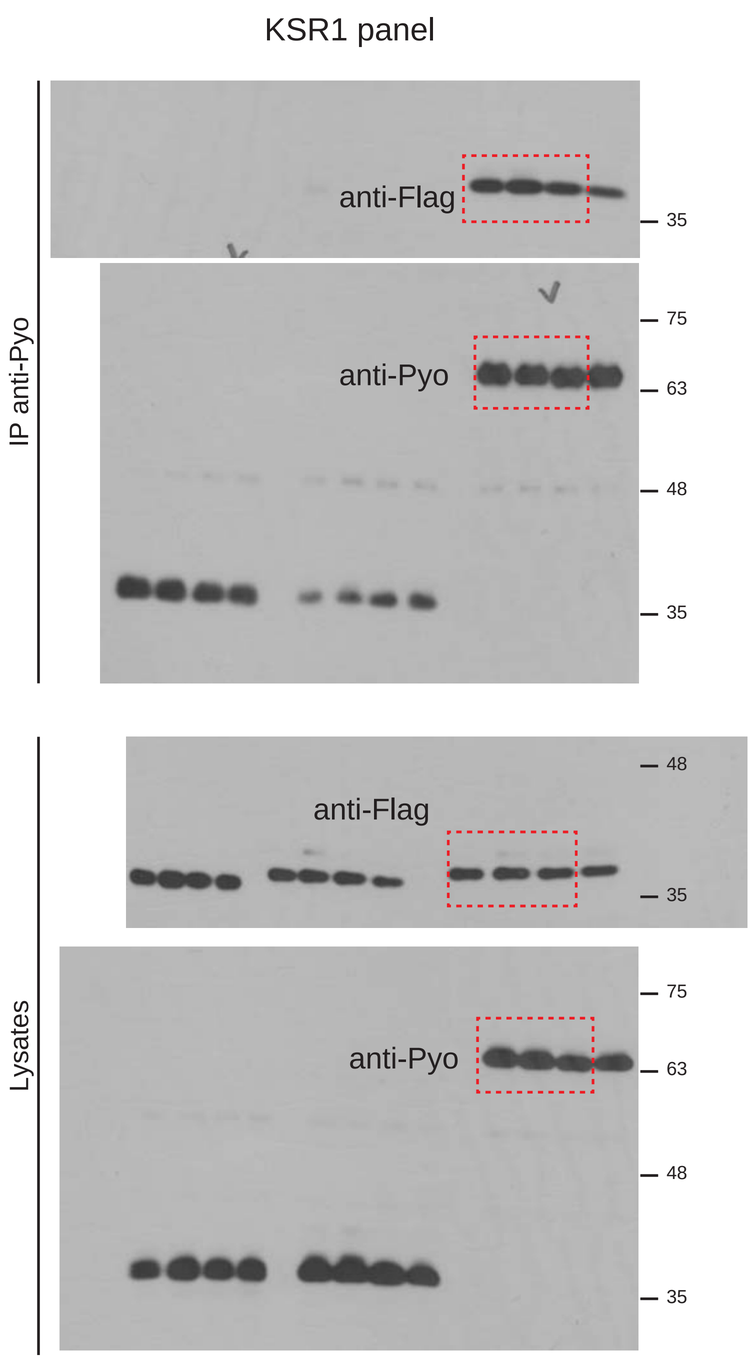

Uncropped immunoblots related to Fig. 4b

KSR2 panel

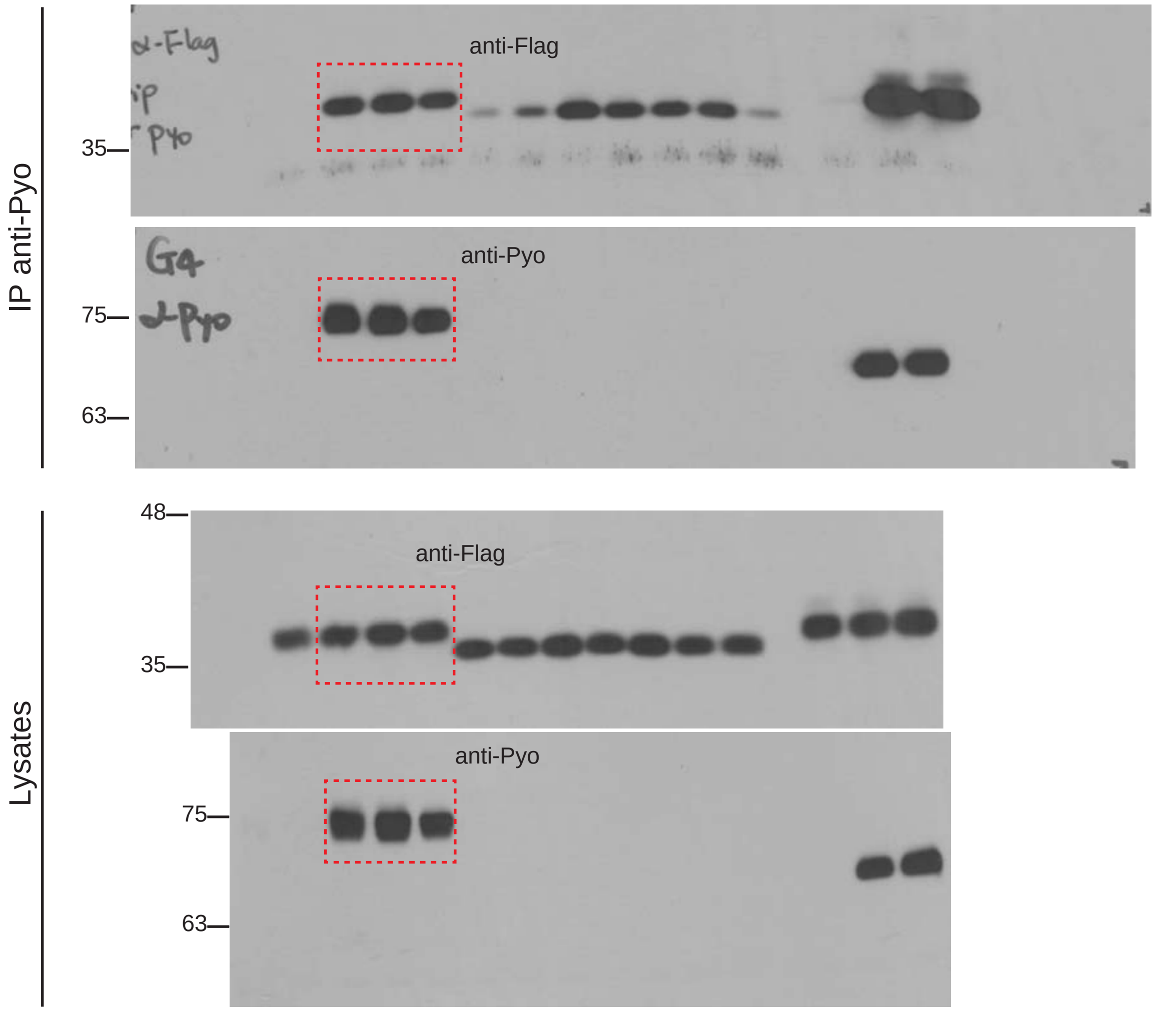

Uncropped immunoblots related to Fig. 4d

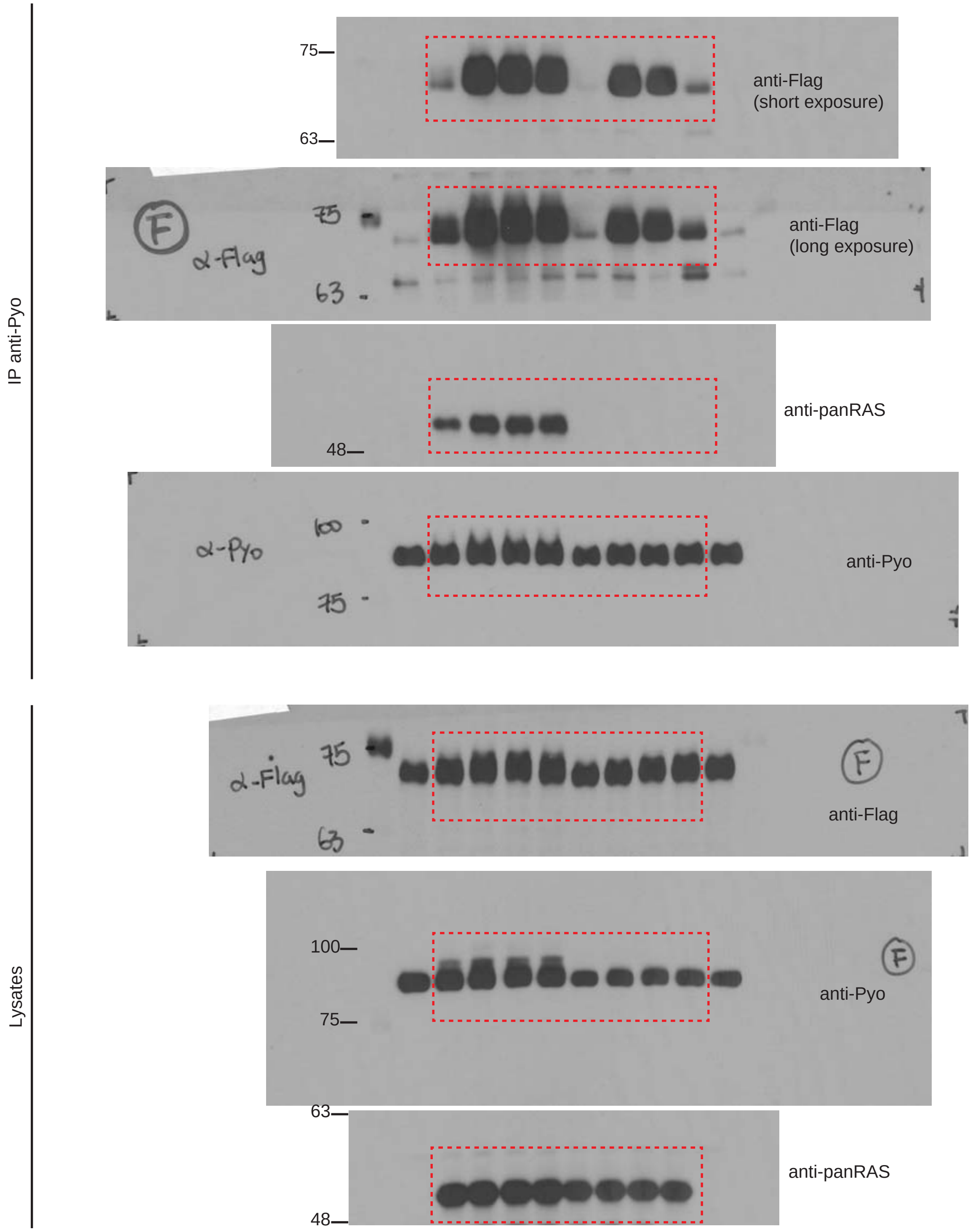

Uncropped immunoblots related to Fig. 5c

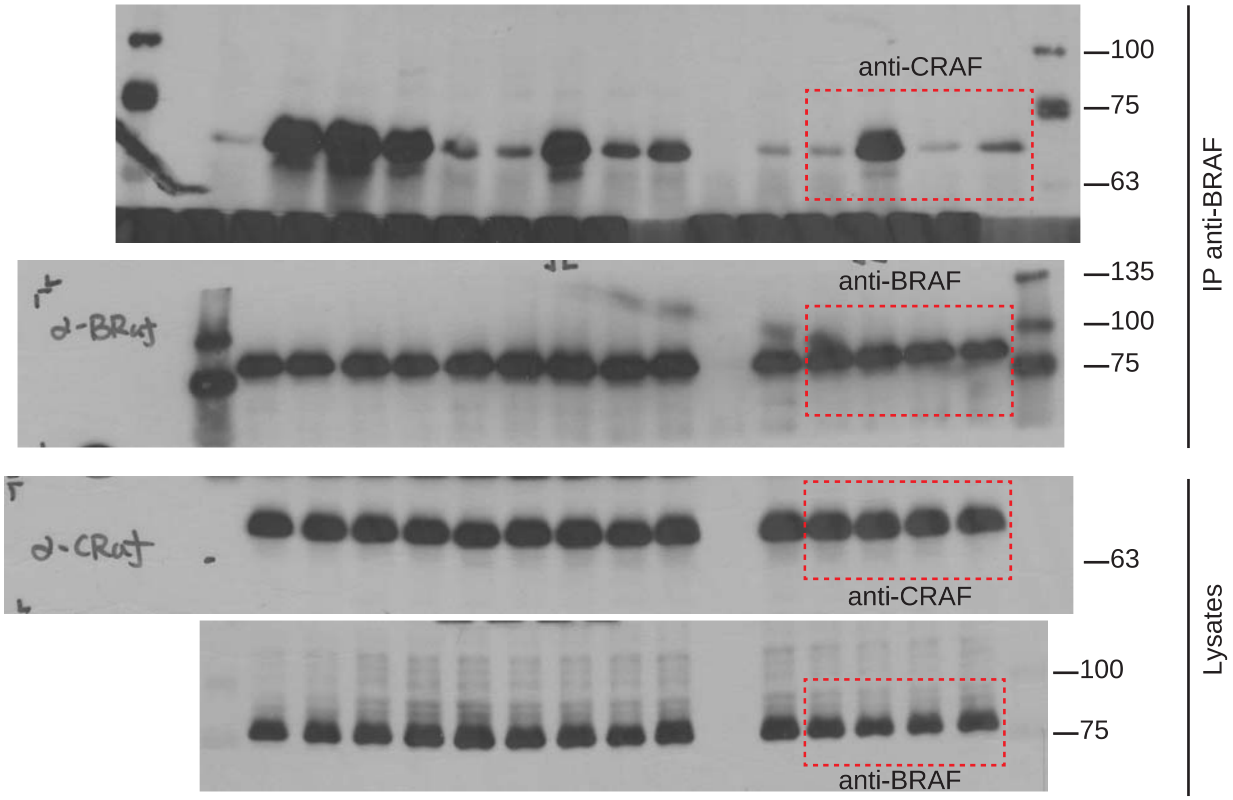

Uncropped immunoblots related to Fig. 5e

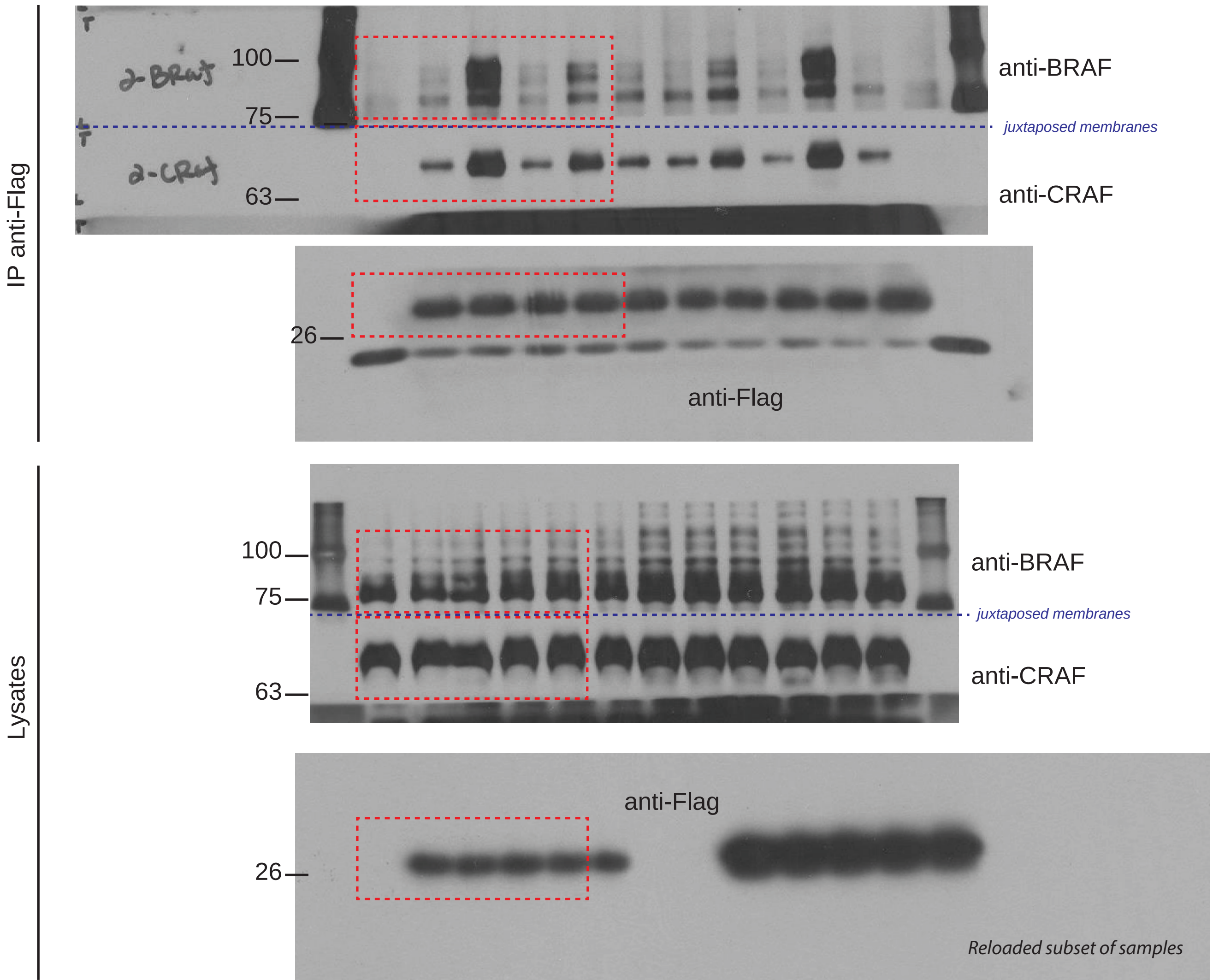

Uncropped immunoblots related to Fig. 5g

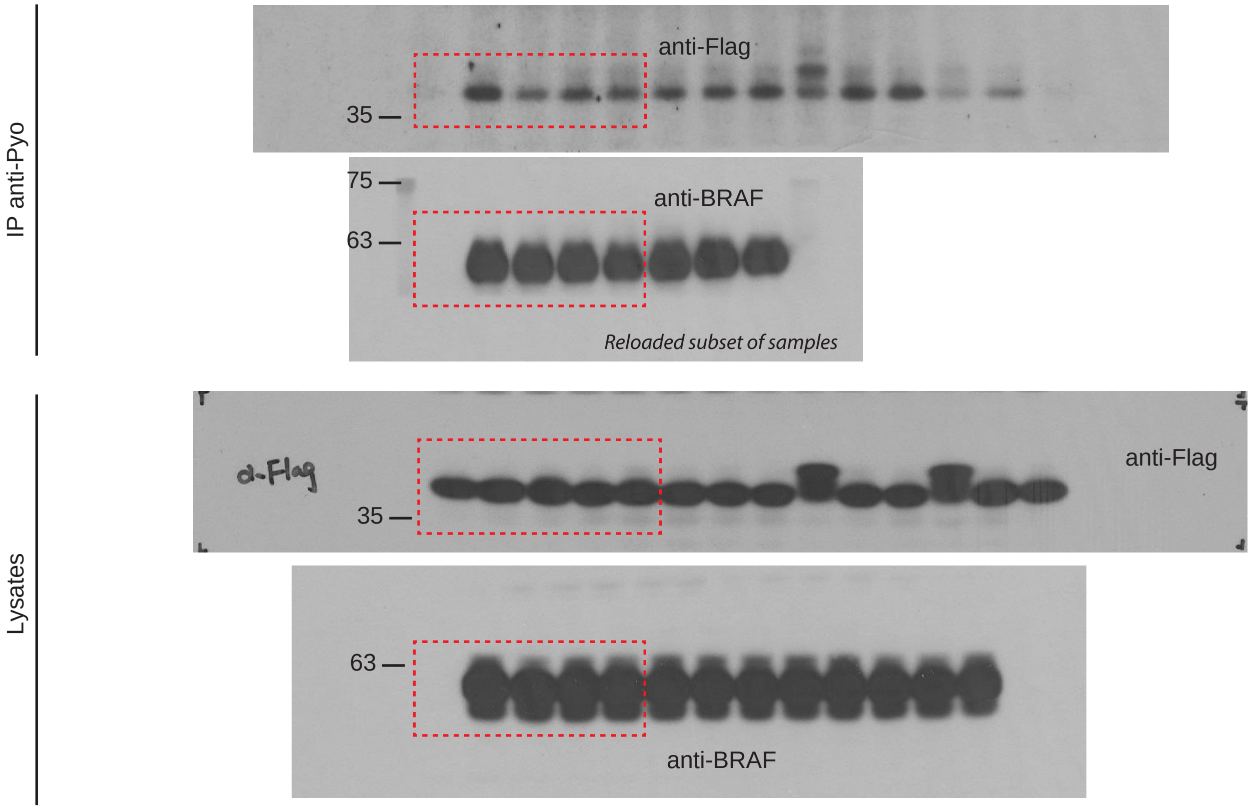

Supplement: Supplementary file 1 — Supplementary Information [file 41467_2017_1274_MOESM1_ESM.pdf]
