# Supplementary material for: RAF inhibitors promote RAS-RAF interaction by allosterically disrupting RAF autoinhibition
Source: Nat Commun. 2017 Oct 31;8:1211. doi: 10.1038/s41467-017-01274-0 (PMC5662619; doi:10.1038/s41467-017-01274-0)
Supplement: Supplementary file 3 — Descriptions of Additional Supplementary Files [file 41467_2017_1274_MOESM3_ESM.pdf]

Description of Additional Supplementary Files

File Name: Supplementary Dataset 1
Description: Binding and BRET data for representative RAF inhibitors. Each inhibitor was tested in a TR-FRET displacement assay using recombinant BRAF kinase domain. BRET EC50 and YMAX are presented for each inhibitor in the following assays: BRAF-BRAF dimerization, KRASG12V-BRAF interaction, BRAFNTR-BRAFKD interaction, and KRASG12V-CRAF interaction. Means and standard deviations from at least three independent repeats are listed.

File Name: Supplementary Dataset 2
Description: Compounds used in this study.
